# A Novel Active Rehabilitation Model for Stroke Patients Using Electroencephalography Signals and Deep Learning Technology

**DOI:** 10.3389/fnins.2021.780147

**Published:** 2021-10-28

**Authors:** Ming Gao, Jie Mao

**Affiliations:** ^1^College of Sports Science and Technology, Wuhan Sports University, Wuhan, China; ^2^College of Sports Engineering and Information Technology, Wuhan Sports University, Wuhan, China

**Keywords:** stroke, rehabilitation model, deep learning technology, deep neural network model, EEG recognition

## Abstract

The main clinical manifestations of stroke are motor, language, sensory, and mental disorders. After treatment, in addition to being conscious, other symptoms will still remain in varying degrees. This is the sequelae of stroke, including numbness, facial paralysis, central paralysis, and central paralysis. If the sequelae of stroke are not treated effectively, they can easily develop into permanent sequelae. Most of the affected people have sequelae, and most of them have symptoms of upper limb paralysis. Therefore, it is of great significance to study how to carry out effective rehabilitation training for stroke patients to reduce the disease and even restore their motor function. Based on this background, this research aims to use deep learning technology to design a stroke rehabilitation model based on electroencephalography (EEG) signals. First, the patient’s EEG signal will be preprocessed. Then, an improved deep neural network model (IDNN) is used to get the EEG classification results. The traditional DNN model construction process is simple and suitable for scenarios where there is no special requirement for the data format, but the generalization of a single DNN model is usually poor. Large margin support vector machine (LM_SVM) is an extension method of support vector machine (SVM), suitable for any occasion. By optimizing the edge distribution, better generalization performance can be obtained. Taking into account the advantages of DNN and LM_SVM and the high aliasing characteristics of stroke data, an improved DNN model is proposed. Finally, based on the EEG recognition result of the model, the rehabilitation equipment is controlled to assist the patient in rehabilitation treatment. The experimental results verify the superiority of the EEG classification model used, and further prove that this research has good practical value.

## Introduction

Stroke is a disease that causes brain dysfunction due to blockage or rupture of blood vessels in the brain. According to the cerebral blood circulation disorder, it is divided into two types: hemorrhagic type and ischemic type. Most stroke patients are ischemic, that is, the softening and necrosis of local brain tissue due to blood circulation, ischemia, and hypoxia. There are more ischemic strokes, accounting for about 80% of all strokes (Liu and Chen, 2014). According to the statistics of the World Health Organization, stroke has become one of the main diseases of human death and disability worldwide (Sheorajpanday et al., 2011; Walter et al., 2016). The age of patients tends to be younger. Due to the different extent of damage to the affected brain regions and tissues, the symptoms of stroke also vary. Symptoms include paralysis of limbs, impaired language and communication skills, loss of comprehension, and loss of emotional thinking. Among them, post-stroke cognitive dysfunction (PSCI) is a common complication (Pendlebury et al., 2015). According to different races, regions and diagnostic criteria, the incidence of PSCI in Europe and America is 20∼80% (Douiri et al., 2013; Sun et al., 2014).

In recent years, with the emergence of new treatment methods such as computer-assisted cognitive training, new drug development, and occupational therapy, it has brought hope to stroke rehabilitation. These treatment methods have improved the quality of rehabilitation, shortened the recovery period, and reduced the cost of rehabilitation (Lin et al., 2014). Topping (2002) developed a robot that can assist children with cerebral palsy to eat. This is an assistive robot, and its upgraded version can also provide services such as cleaning and brushing. Krebs et al. (1998) has developed a robot that can assist rehabilitation training based on brain nerves. This robot can perform upper limb rehabilitation training for stroke patients. Compared with traditional therapy, it has achieved good results. Leigh et al. (2006) collects EEG by implanting a chip in the sample brain. The samples can realize a series of behaviors in daily life through the brain-computer interface, such as checking emails, turning on the TV, controlling the damaged upper limbs to grasp and so on. This is of great significance to the research direction of using ideas to control external devices, and it also provides new ideas for rehabilitation training based on brain-computer interface technology. Gomez-Rodriguez et al. (2011) developed a rehabilitation system based on motor imagination. The system first records the EEG signals when the sample imagines the arm movement, and then recognizes the movement tendency of the sample online, and drives the robotic arm to move based on the recognition result. Based on the existing research, it can be seen that the rehabilitation of stroke mainly relies on medical rehabilitation equipment. In order to assist patients in active rehabilitation training, some active rehabilitation methods have been proposed. The core idea of this method is to control the rehabilitation equipment based on the patient’s EEG signal recognition result, and then assist the patient to train their consciousness and limbs synchronously. Gert et al. (2003) designed a MI-EEG-based rehabilitation system for the recovery of patients’ arm motor function.

In the rehabilitation research of stroke samples, the most important thing is to classify and recognize EEG signals. The classification accuracy of EEG directly determines the rehabilitation effect. EEG recognition research is mainly divided into two categories, which are based on machine learning (Jiang et al., 2019, 2021) and deep learning (Elman, 1990; Dahl et al., 2012). The core idea of EEG recognition based on machine learning is to preprocess the collected EEG signals, then feature extraction, and finally use the trained classifier to recognize feature data to obtain the classification results. The commonly used feature extraction methods in this process are time domain analysis method (Fingelkurts et al., 2007), frequency domain analysis method (Anderson et al., 1995), time-frequency domain analysis method (Zandi et al., 2010), and so on. Commonly used classifiers are SVM (Garrett et al., 2003), Linear Discriminant Analysis (LDA; Cortes and Vapnik, 1995), BP neural network (Williams et al., 1986). The core idea of EEG recognition based on deep learning is to use various deep learning algorithms to directly classify EEG signals. Commonly used deep learning models are Deep Neural Networks (DNN; Ghonchi et al., 2020), Deep Belief Networks (DBN; Leigh et al., 2006), Auto Encoder (AE; Li et al., 2021), Spare Coding (SC; Tokuhiro, 2000), Convolutional Neural Networks (CNN; Deng et al., 2009) and Recurrent Neural Network (RNN; Petrosian et al., 2000), Long Short-Term Memory (LSTM; Hochreiter and Schmidhuber, 1997).

Deep neural network uses the output of the previous layer as the input of the next layer to obtain the hierarchical characteristics of the input data. Finally, by adjusting the parameters in the neural network layer, the difference between the output and the input is minimized. The DNN model construction process is relatively simple and is suitable for scenarios where there is no special requirement for the data format, but usually a single DNN model has poor generalization. LM_SVM is suitable for any occasion based on SVM. It obtains better generalization performance by optimizing the edge distribution. The minimum margin of LM_SVM is usually smaller than that of SVM, and the margin distribution is better than SVM. In most cases, LM_SVM usually produces a larger margin than SVM. By maximizing the edge mean and variance at the same time, LM_SVM can effectively process data with high aliasing. Considering the advantages of DNN and LM_SVM and the high aliasing characteristics of stroke data, an improved DNN (IDNN) is proposed. The EEG signal recognition result based on IDNN can effectively control the rehabilitation equipment. The core work of this research is:

(1) An improved DNN model is proposed: This model uses DNN as the kernel function of LM_SVM to perform kernel mapping on the input data. Then the output of DNN is fed back to LM_SVM, and LM_SVM outputs the final classification result of the model. IDNN makes up for the shortcomings of using DNN alone, so that the constructed model has better generalization. As the core part of LM_SVM, the kernel function directly affects the classification performance of the model. In this study, DNN is used as a kernel function to solve the problem of kernel selection.
(2) EEG recognition is based on the IDNN model: Compared with the classification results of other models, the model used has a higher recognition rate. Applying EEG recognition results to rehabilitation training equipment for stroke, patients can control the rehabilitation equipment to complete corresponding actions more quickly and accurately. This rehabilitation training model can not only train the thinking but also assist in the completion of the movement of the limbs, and has good practical value.

## Related Work

### Stroke Rehabilitation Treatment Process

At present, China has established a three-level rehabilitation system for rehabilitation of stroke samples. The difference between each level of rehabilitation lies mainly in the distinction of rehabilitation sites. “First-level rehabilitation” refers to early rehabilitation treatment and conventional treatment received in the emergency room or neurology ward. The training of the ability to sit down and the recovery of eating ability lays the foundation for the sample to leave the stroke ward for upgraded rehabilitation. “Secondary rehabilitation” means that the sample enters the rehabilitation ward or rehabilitation center. At this stage, clinical treatment is used to assist rehabilitation treatment, mainly for rehabilitation treatment. The main purpose of this stage is to improve the sample’s physical ability and daily living ability. After training at this stage, most samples can take care of themselves and return to their families. “Tertiary rehabilitation” refers to the treatment in which the sample leaves the hospital and returns to the community or home. Through further daily life and sports training to consolidate recovery.

In “first-level rehabilitation,” most of the samples are in a coma or bedridden state, and only basic early recovery is possible. Rehabilitation treatment is less involved. Rehabilitation treatment mainly includes rehabilitation care, recovery of consciousness, management of swallowing function, and passive activities with the help of others. In the “three-level rehabilitation,” the place of rehabilitation is changed from a hospital to a home or a community, and the practitioners of auxiliary treatment are physicians. This study is mainly used for the “secondary rehabilitation” treatment stage where rehabilitation treatment is more involved and the location is the rehabilitation center.

**FIGURE 1:**
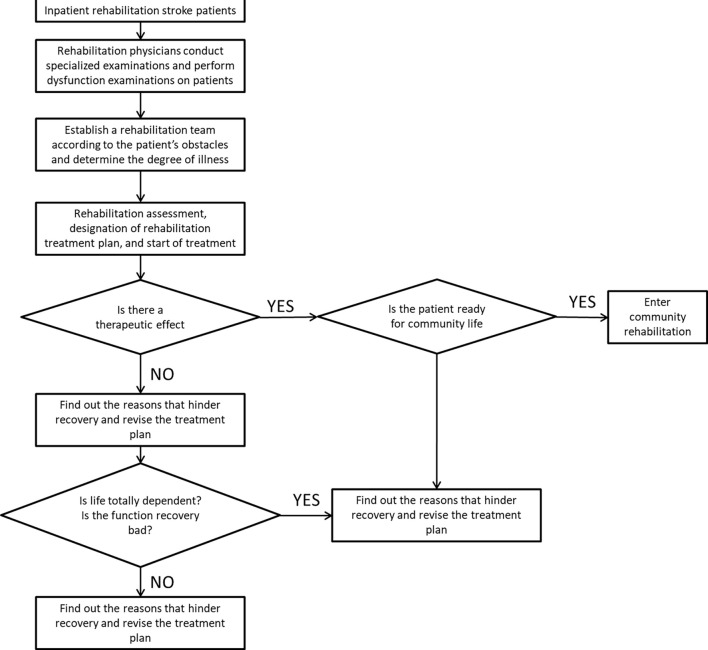
Secondary rehabilitation process for stroke.

The secondary rehabilitation process for stroke is shown in Figure 1. After the sample is transferred to the rehabilitation center or the rehabilitation department of the hospital, the rehabilitation physician collects the relevant medical history of the patient and conducts specialized examination and functional screening of the patient. Mainly check the degree of obstacles in motor function, sensory function, cognitive communication, and self-care ability in life. Determine the composition of the rehabilitation team based on the dysfunction. Further inspection of the samples to determine the degree of disease and staging results. Then the patients are evaluated for rehabilitation, and rehabilitation treatment plans are developed and treatments are carried out. The training content at this stage is mainly for the training of daily life functions such as sitting, standing and walking, eating and dressing, and going up and down stairs. After a period of training, evaluate the rehabilitation effect of the sample. If the sample’s dysfunction has improved, it can enter the community for rehabilitation. If the patient does not meet the return to community living standards, it is recommended to continue to be hospitalized for rehabilitation.

### Deep Neural Network

**FIGURE 2:**
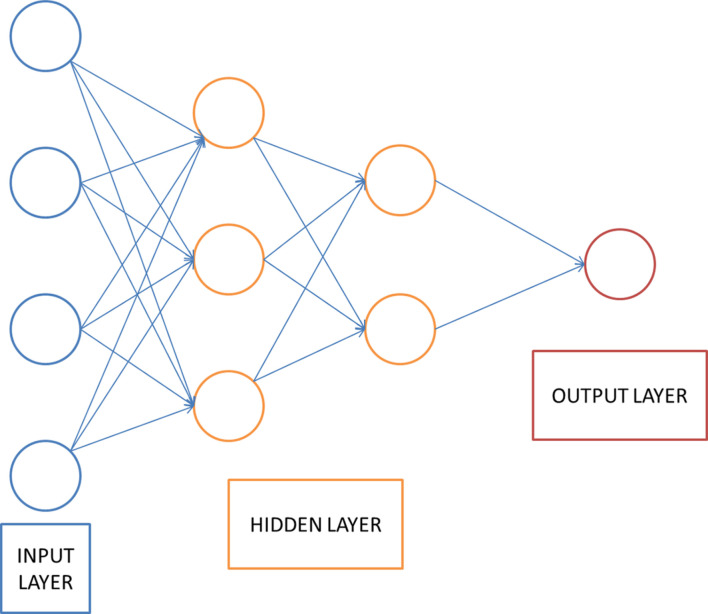
DNN structure.

This section introduces the related principles of the neural network model involved in the stroke assisted rehabilitation model mentioned. DNN is a feed-forward artificial neural network that contains multiple hidden layers between the input and output layers. Compared with ordinary neural networks, deep learning networks require unsupervised learning before supervised learning, and then use the learned weights as initial values for supervised learning for training. This supervised learning helps reduce the risk of overfitting. The DNN model structure is shown in Figure 2.

The DNN shown in Figure 2 uses a multi-layer neural network to simulate the human brain to learn features automatically. Extract features from the original data to analyze the data. The basic idea of deep learning is to use a multi-layer neural network, and the output of the previous layer is used as the input of the next layer. Based on this method, the hierarchical representation of the input data is realized, and the hierarchical characteristics of the input data are obtained. Finally, by adjusting the parameters in the neural network layer, the difference between the output and the input is as small as possible. The implementation steps of DNN are described as follows:

(1) Unsupervised learning is used to pre-train each layer of the network;
(2) Based on the unsupervised learning training of each layer of neural network, the training results obtained will be used as the input of the next layer;
(3) A top-down supervision algorithm is used to adjust the parameters of the neural network layer.

Since deep learning was proposed, it has attracted the attention of many researchers. After more than 10 years of development, deep learning technology has been widely used in many fields of data mining. Many impressive results have been achieved in pattern recognition, intelligent robots, automatic control, predictive estimation, biology, medicine, and economics.

Video behavior recognition currently exists in algorithms, both manual feature-based, and deep learning-based methods, to extract spatial and temporal information from the video, spatial information contains information about the appearance of the video, and temporal information contains information about the temporal continuity between frames of the video. The spatial information is usually extracted using a convolutional neural network, and the temporal information is usually extracted using a cyclic neural network, which uses different filters to get different picture features. Different convolutional kernels correspond to different picture effects.

### LM_SVM

LM_SVM is an extension method of SVM, which is suitable for any occasion that supports SVM. LM_SVM obtains better generalization performance by optimizing the edge distribution. The minimum margin of LM_SVM is usually larger than that of SVM, and the margin distribution is better than SVM. Therefore, compared to SVM, LM_SVM has more advantages in processing data with high aliasing. The SVM algorithm based on edge theory introduces the mean value of edge distribution features on the basis of SVM. The marginal distribution feature of LM_SVM is the first-order statistics of marginal samples, which can be expressed as:


(1) $$\bar{\gamma }=\frac{1}{N}\sum_{i=1}^{N}y_{i}\left(\varphi^{T}\left(x_{i}\right)w+b\right)=\frac{1}{N}{\left(Xy\right)}^{T}W$$

LM_SVM needs to ensure to maximize the first-order statistics of the edge samples. Therefore, by introducing variables C and *ξ*_*i*_, the objective function of LM_SVM is given as:


(2) $$\min_{w,b}\frac{1}{2}{||W||}^{2}+C\sum_{i=1}^{N}\xi_{i}^{2}-\lambda \bar{\gamma }$$

(3) $$\begin{array}{c} s.t.y_{i}\left(\varphi^{T}\left(x_{i}\right)w+b\right)\geq 1-\xi_{i}\\ \xi_{i}\geq 0,i=1∼N \end{array}$$

## Improved Deep Neural Network-Based Rehabilitation Model

### Execution Process of Rehabilitation Model Based on Improved Deep Neural Network

**FIGURE 3:**
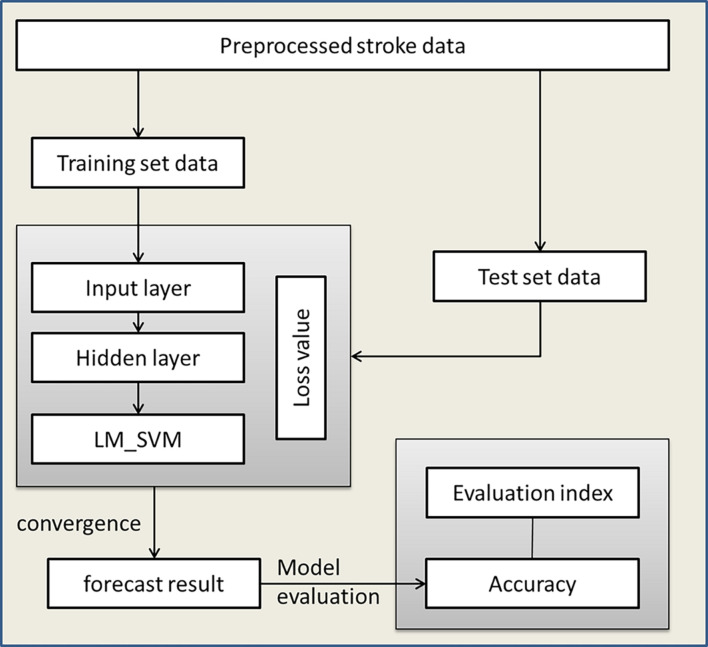
Rehabilitation model execution process.

Aiming at the advantages of DNN and LM_SVM and the characteristics of high aliasing of stroke data, this paper proposes an IDNN model. This model uses DNN as the kernel function of LM_SVM to perform kernel mapping on the input data. Then the output of DNN is fed back to LM_SVM, and LM_SVM outputs the final classification result. IDNN makes up for the shortcomings of using DNN alone, so that the constructed model has better generalization. As the core part of LM_SVM, the kernel function directly affects the classification performance of the model. The execution process of the rehabilitation model based on IDNN is shown in Figure 3.

### Improved Deep Neural Network Model

**FIGURE 4:**
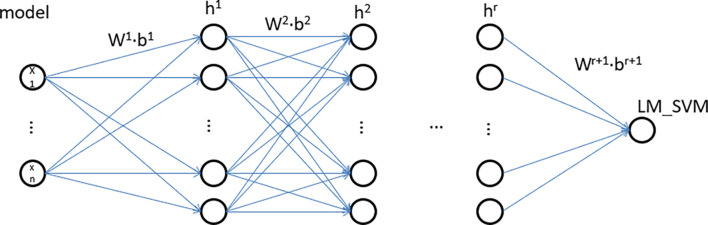
IDNN structure.

The IDNN model is mainly composed of two parts, namely DNN and LM_SVM. The DNN model of IDNN consists of an input layer and several hidden layers. Compared with other neural network models, IDNN has no output layer, and its output layer is replaced by LM_SVM. The number of hidden layers of IDNN and the number of neurons in each layer need to be determined based on experiments. LM_SVM maximizes the average value of the edge vector in the process of establishing the SVM, and separates the support vector of the SVM as much as possible. Figure 4 depicts an IDNN structure with r hidden layers, and shows how the data changes in the model. In this model, DNN expresses the kernel mapping as an explicit function through a fully connected network. It can not only solve the kernel function selection problem of LM_SVM, but also learn more effective stroke data features, thereby improving classification performance. The output of the model is used as the input of LM_SVM, and the final classification output is completed by LM_SVM.

The general DNN model, like the perceptron, is composed of a linear relationship *z* = ∑*w*_*i*_*x*_*i*_ + *b* and an activation function σ(*z*). The IDNN model proposed in this study can be regarded as a deep neural network model whose output layer is LM_SVM. Define the linear coefficient from the *k*th neuron of the *h*-1th layer to the *j*th neuron of the *h*th layer as $w_{jk}^{t}$. Accordingly, the bias for defining the *j*th neuron of the *i*th layer is defined as $b_{j}^{i}$, and the output is $a_{j}^{i}$. For example, $w_{32}^{2}$ represents the linear coefficient from the third neuron of the second layer to the second neuron of the second layer, and the third neuron of the second layer is denoted as $b_{3}^{2}$. If there are a total of *N* neurons in the *h*-1th layer and *n* neurons in the *h*th layer, the linear coefficients of the *l*th layer can be expressed as an *c*^∗^*m* matrix. Correspondingly, the bias matrix of the *h* layer can be expressed as an *c*^∗^1 vector *b^h^*, the output of the first layer is an *c*^∗^1 vector *a^h^*, and the input layer’s non-linearity coefficients and bias terms. Based on the above analysis, the output formula of layer *h* is:


(4) $$a^{h}=\sigma \left(Z_{t}\right)=\sigma \left(W^{h}a^{t-1}+b^{h}\right)$$

Eq. (3) is the forward propagation expression of DNN. In the DNN model, the activation function of the output layer usually adopts softmax. Since the classification output result of DNN has an error with the actual result, it is necessary to calculate the error between the category value of the output layer and the actual value. And the error is propagated back from the output layer to the hidden layer until it propagates to the input layer. In the back propagation process, the values of various parameters are adjusted according to the error. Continue to iterate the above process until the model converges. The mean square error is often used to measure loss. The loss function of each data is as follows:


(5) $$J=\frac{{||a^{H}-y||}_{2}^{2}}{2}$$

Where *a^H^* represents the classification value of different categories, and *y* represents the actual value of different categories. Gradient descent strategy is used to solve the *w* and *b* of each layer. The loss function of the output layer can be expressed as:


(6) $$J=\frac{{||a^{H}-y||}_{2}^{2}}{2}=\frac{{||\sigma \left(W^{H}a^{H-1}+b^{H}\right)-y||}_{2}^{2}}{2}$$

The gradient calculation formula for solving *w* and *b* is as follows:


(7) $$\frac{\partial J}{\partial W^{H}}=\frac{\partial J}{\partial Z^{H}}\frac{\partial Z^{H}}{\partial W^{H}}=\left(a^{H}-y\right)⊙\sigma \left(Z^{H}\right)\left(Z^{H-1}\right)$$

(8) $$\frac{\partial J}{\partial b^{H}}=\frac{\partial J}{\partial Z^{H}}\frac{\partial Z^{H}}{\partial b^{H}}=\left(a^{H}-y\right)⊙\sigma^{\prime }\left(Z^{H}\right)$$

_⊙_ stands for Hadamard product. Let $\delta^{H}=\frac{\partial J}{\partial Z^{H}}$, δ^*H*^ is the gradient, the gradient of the output layer is:


(9) $$\delta^{H}=\frac{\partial J}{\partial Z^{H}}=\frac{\partial J}{\partial Z^{H}}\frac{\partial Z^{H}}{\partial Z^{H-1}}\cdots \frac{\partial Z^{H+1}}{\partial Z^{H}}$$

From Eq. (5), the following expression is obtained:


(10) $$Z_{h}=W^{h}a^{h-1}+b^{h}$$

Therefore, the gradients of *w* and *b* in the *H*-th layer can be expressed as:


(11) $$\frac{\partial J}{\partial W^{h}}=\frac{\partial J}{\partial Z^{h}}\frac{\partial Z^{h}}{\partial W^{h}}=\delta^{h}{\left(a^{h-1}\right)}^{T}$$

(12) $$\frac{\partial J}{\partial b^{h}}=\frac{\partial J}{\partial Z^{h}}\frac{\partial Z^{h}}{\partial b^{h}}=\delta^{h}$$

From mathematical induction, the recurrence relation of δ^*h*^ can be obtained as follows:


(13) $$\delta^{h}=\delta^{h+1}\frac{\partial Z^{H+1}}{\partial Z^{H}}={\left(W^{h+1}\right)}^{T}\delta^{h+1}⊙\sigma \left(Z^{h}\right)$$

In summary, the back propagation process of IDNN is summarized as follows:


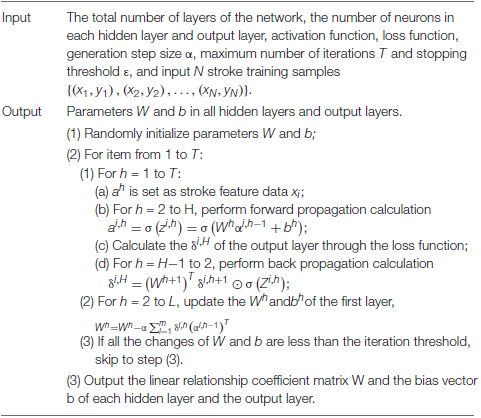


## Simulation Experiment and Analysis

### Experimental Data

**FIGURE 5:**
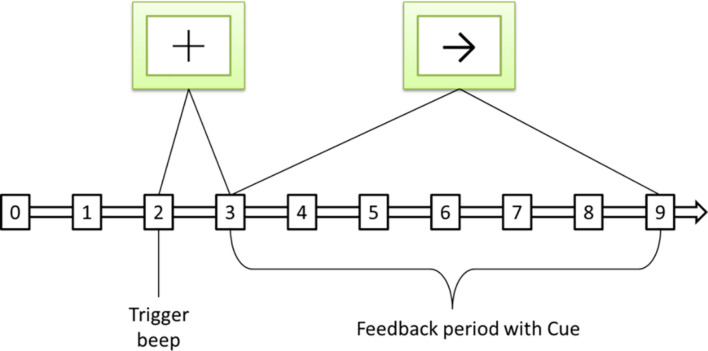
Sequence diagram of a single motor imaging task. The symbol + represents the start signal, and the symbol → represents the directional signal.

This article uses the BCI Competition II dataset III sports imagination data set. The process of generating the data set is as follows: 280 left and right hand motor imagination EEGs of an adult female with normal physical indicators in all aspects are collected. Randomly select half of the data from the 280 times as the training set, and the remaining general data as the test set. The left and right hand imagination data in the training set each account for half. The sampling frequency of 128 Hz is used when collecting EEG, and the bandpass filter is 0.5∼30 Hz. The process of each data collection is shown in Figure 5.

The total time used in each experiment is 9 s. The preparation time of 2 s, at 3 s, prompts that the sample is about to perform the motor imaging task, and the screen displays the “+” symbol at the same time, and the process duration is 1 s. In the 4th second, an arrow appears on the screen, and the sample moves in the direction indicated by the arrow by dragging the feedback bar through motion imagination, and keeps it until the end of the 9th second.

### Experimental Environment

This article uses the accuracy shown in Eq. (13) as the evaluation index for different classification models. Among them, TP represents the number of samples that are classified as positive and are actually positive. TN represents the number of samples that are classified as negative, but are actually negative. FP represents the number of samples classified as positive but actually negative. FN represents the number of samples classified as negative but actually positive.


(14) $$A\text{cc}=\frac{TP+TN}{TP+TN+FN+FP}$$

The experimental environment is shown in Table 1.

**TABLE 1:** Experimental environment.

**Hardware**	
Processor	RAM
Intel(R) Xeon(R) CPU E5-2650 V2@ 2.60 GHz* 4	8 GB
**Software**	
Operating system	Simulation environment
Microsoft Windows 7 (64-bit)	Python 3.6

*Represents the multiplication.

### Experimental Results and Analysis

#### Determination of Experimental Parameters

The experimental parameters that need to be confirmed during the experiment are the number of hidden layers of the network model and the number of neurons in each layer, the number of model iterations, the learning rate, and the λ value. In order to determine the structure of the network, the number of hidden layers and the number of neurons in each layer need to be determined. The experimental results of different hidden layer numbers and unit numbers are shown in Table 2. The number of iterations is 2,500.

**TABLE 2:** Classification accuracy of different numbers of neurons in each layer.

Hidden layer number\number of neurons	5	6	7	8	9	10	11	12
Layer 1	0.9213	0.9316	0.9320	0.9239	0.9418	0.9567	0.9583	0.9512
Layer 2	0.9522	0.9661	0.9587	0.9345	0.9465	0.9407	0.9323	0.9293
Layer 3	0.9405	0.9584	0.9509	0.9423	0.9486	0.9463	0.9394	0.9320
Layer 4	0.9373	0.9340	0.9329	0.9406	0.9234	0.9321	0.9290	0.9256
Layer 5	0.9251	0.9386	0.9318	0.9257	0.9265	0.9286	0.9255	0.9228

**FIGURE 6:**
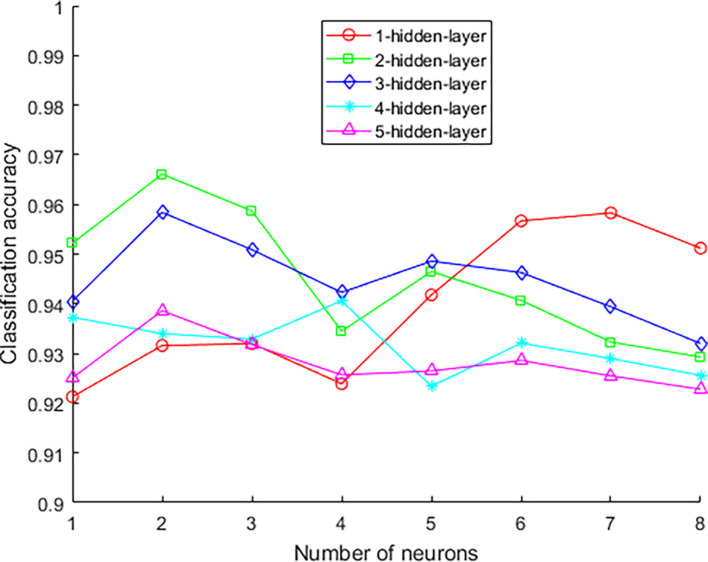
The accuracy rate changes with the number of hidden layers.

The best classification accuracy is obtained when the hidden layer 1 contains 11 neural units, so the number of selected units in the first layer is 11. The classification accuracy is the best when the hidden layers 2, 3, and 5 contain 6 neural units, so the number of selected units in the first layer is 6. The best classification accuracy is obtained when the hidden layer 4 contains 8 neural units, so the number of selected units in the first layer is 8. On the other hand, as the number of hidden layers increases, the accuracy rate decreases from the original 0.9583 to 0.9386, and its changing trend is shown in Figure 6. This trend shows that it is not that the more hidden layers, the better the model performance. When the number of hidden layers is increased, the model construction time becomes significantly longer, but the accuracy rate begins to decrease. Therefore, considering the above factors comprehensively, this paper selects a network with two hidden layers and a combination of neurons in each layer (11, 6) as the final stroke rehabilitation model.

The difference in learning rate will also affect the performance of the network model. The learning rate range selected in this paper is 0.05–0.14, and the step size is 0.01. The experimental results of different learning rates are shown in Table 3. The data in Table 3 shows that when the learning rate is 0.07, the model has the best accuracy. Therefore, the learning rate in the experiment is 0.07.

**TABLE 3:** The effect of learning rate on accuracy.

Learning rate	0.05	0.06	0.07	0.08	0.09	0.1	0.11	0.12	0.13	0.14
Acc	0.9564	0.9612	0.9658	0.9451	0.9472	0.9360	0.9452	0.9401	0.9359	0.9363

Set the learning rate to 0.78, the value range of λ is 0.01–100, and the experimental results obtained are shown in Table 4. According to the data in Table 4, the final selected value of λ is 0.1.

**TABLE 4:** The influence of λ value on accuracy.

_λ_	0.01	0.05	0.1	0.5	1	5	10	50	100
Acc	0.9545	0.9562	0.9660	0.9621	0.9556	0.9487	0.9506	0.9442	0.9465

#### Performance Comparison of Different Models

The above experiment determined the number of hidden layers and the corresponding number of neurons, learning rate and *A* value. Based on these parameter settings, the structure of the network model used in this article is basically determined. In order to evaluate the effectiveness of the model used, the selected comparison models are BP neural network (Liu, 2019), DNN (Sun et al., 2021), CNN (Moon et al., 2020), LSTM (Wang et al., 2018). The parameter settings of each network are the same as the corresponding references. Since rehabilitation training requires relatively high timeliness, the running time of different models is also an important indicator. Table 5 shows the recognition accuracy and running time comparison of different models on the data set.

**TABLE 5:** Performance comparison of each model.

Index\Model	BP	CNN	DNN	LSTM	IDNN
Acc	0.9139	0.9443	0.9596	0.9611	0.9658
Time(s)	74	109	113	132	120

In terms of accuracy, the IDNN model used in this article has the highest accuracy. It shows that DNN expresses the kernel mapping as an explicit function through a fully connected network, which can not only solve the kernel function selection problem of LM_SVM, but also learn more effective stroke data features, thereby improving classification performance.

In terms of running time, the BP model takes significantly less time than other deep learning models. This fully shows that the deep learning model needs further improvement in terms of time complexity. The running time of the model used is not the least among other models of the same type, only less than LSTM. This shows that on the basis of improving the accuracy of the model used, a certain amount of time complexity has been added, but the added time is not much.

## Conclusion

In order to better assist the rehabilitation training of stroke samples, this article uses a rehabilitation model based on deep learning technology. First, the LM_SVM classifier is introduced into the traditional DNN network. LM_SVM improves the generalization performance of the network model by optimizing the edge distribution, thereby improving the accuracy of the network’s classification of EEG. Secondly, the EEG signals of stroke samples are identified based on the IDNN model. Finally, based on the recognition results, the movement of the rehabilitation equipment is controlled to assist the rehabilitation training of stroke samples. Comparative experiments show that the IDNN model proposed has a better classification accuracy for EEG. However, the time complexity has increased, but the increase is not large. In summary, how to reduce the time complexity of the model is the content of this article to continue to study in the future.

## Data Availability Statement

Publicly available datasets were analyzed in this study. This data can be found here: http://www.bbci.de/competition/iii/.

## Author Contributions

MG was mainly responsible for the implementation of the experiment and analysis of the results. JM was mainly responsible for the design of the entire experiment. Both authors contributed to the article and approved the submitted version.

## Conflict of Interest

The authors declare that the research was conducted in the absence of any commercial or financial relationships that could be construed as a potential conflict of interest.

## Publisher’s Note

All claims expressed in this article are solely those of the authors and do not necessarily represent those of their affiliated organizations, or those of the publisher, the editors and the reviewers. Any product that may be evaluated in this article, or claim that may be made by its manufacturer, is not guaranteed or endorsed by the publisher.
